# RETRACTED: Akter et al. Improved Transfer-Learning-Based Facial Recognition Framework to Detect Autistic Children at an Early Stage. *Brain Sci.* 2021, *11*, 734

**DOI:** 10.3390/brainsci16101129

**Published:** 2026-10-20

**Authors:** Tania Akter, Mohammad Hanif Ali, Md. Imran Khan, Md. Shahriare Satu, Md. Jamal Uddin, Salem A. Alyami, Sarwar Ali, AKM Azad, Mohammad Ali Moni

**Affiliations:** 1 Department of Computer Science and Engineering, Jahangirnagar University, Savar, Dhaka 1342, Bangladesh; shilpy339cse@gmail.com (T.A.); hanif_ju03@juniv.edu (M.H.A.); 2 Department of Computer Science and Engineering, Gono Bishwabidyalay, Savar, Dhaka 1344, Bangladesh; imrankhan770707@gmail.com; 3 Department of Management Information Systems, Noakhali Science and Technology University, Sonapur, Noakhali 3814, Bangladesh; shahriarsetu.mis@nstu.edu.bd; 4 Department of Computer Science and Engineering, Bangabandhu Sheikh Mujibur Rahman Science and Technology University, Gopalganj Town Road, Gopalgonj 8100, Bangladesh; jamal.bsmrstu@gmail.com; 5 Department of Mathematics and Statistics, Imam Mohammad Ibn Saud Islamic University, Riyadh 13318, Saudi Arabia; saalyami@imamu.edu.sa; 6 Department of Electrical and Electronics Engineering, University of Rajshahi, Rajshahi 6205, Bangladesh; sarwar1000@gmail.com; 7 School of Biotechnology and Biomolecular Sciences, University of New South Wales, Sydney, NSW 2052, Australia; 8 WHO Collaborating Centre on eHealth, UNSW Digital Health, Faculty of Medicine, University of New South Wales, Sydney, NSW 2052, Australia; 9 Healthy Aging Theme, Garvan Institute of Medical Research, Darlinghurst, NSW 2010, Australia

The journal retracts the article, “Improved Transfer-Learning-Based Facial Recognition Framework to Detect Autistic Children at an Early Stage” [1], cited above.

Following publication, concerns were brought to the attention of the Editorial Office regarding the validity of the underlying dataset used in this article.

In accordance with the journal’s standard procedure, the Editorial Office and Editorial Board conducted an investigation, which verified the concerns related to the methodology and reliability of the third-party dataset utilized in this study. The investigation was also unable to verify that the facial image dataset had been collected with appropriate ethical approval, or that informed consent had been obtained from the legal guardians of the children whose images were included in the dataset.

As the Editorial Office and Editorial Board have determined that the dataset is essential to the findings, they have therefore decided to retract and remove this article, as per MDPI’s retraction policy. This article has been removed in order to protect the identities of those included in the dataset. To maintain the integrity of the scholarly record, the title, author information and this retraction notice will remain permanently available, while the article itself has been removed. This information has also been communicated to relevant indexing services.

This retraction was approved by the Editor-in-Chief of the journal *Brain Sciences*.

The authors have been informed of this retraction and have indicated their disagreement with this decision.
